# LINE-1 CpG methylation in canine blood and cortical DNA tracks epigenetic changes with age

**DOI:** 10.1007/s11357-026-02429-y

**Published:** 2026-07-27

**Authors:** Dávid Jónás, Kitti Tátrai, Balázs Egyed, Eniko Kubinyi

**Affiliations:** 1 Department of Ethology, ELTE Eötvös Loránd University, Pázmány Péter Sétány 1/C, Budapest, 1117 Hungary; 2 MTA-ELTE Lendület “Momentum” Companion Animal Research Group, Pázmány Péter Sétány 1/C, Budapest, Hungary; 3 Department of Genetics, ELTE Eötvös Loránd University, Pázmány Péter Sétány 1/C, Budapest, 1117 Hungary; 4 ELTE NAP Canine Brain Research Group, Budapest, Hungary

**Keywords:** Dog, Aging, Cytosine methylation, LINE-1, Cell-free DNA

## Abstract

Aging is accompanied by progressive epigenetic alterations, including changes in DNA methylation affecting both gene-associated regions and repetitive elements such as LINE-1 retrotransposons. Reduced epigenetic repression of transposable elements has been implicated in age-related genomic instability. Circulating cell-free DNA (cfDNA) provides a minimally invasive substrate for monitoring systemic molecular changes; however, age-related LINE-1 methylation dynamics in cfDNA remain poorly characterized in non-human species. Dogs represent a valuable translational model for aging research due to their shared environment with humans and shorter lifespan. We investigated age-associated LINE-1 CpG methylation in plasma-derived cfDNA and in prefrontal cortex genomic DNA from dogs. Sequence analysis of 264 potentially active canine LINE-1 elements identified a highly conserved central region spanning ORF1 and ORF2, with the targeted CpG site located within a locally low-variability segment, supporting multi-copy assessment. Methylation was quantified using methylation-sensitive restriction endonuclease digestion followed by real-time quantitative PCR (MSRED-PCR), with bisulfite conversion–based PCR (BSC-PCR) used for technical validation. In clinically healthy dogs, LINE-1 methylation in blood cfDNA showed a strong negative association with chronological age, best described by a nonlinear (logarithmic) model. In prefrontal cortex samples, older dogs exhibited significantly lower LINE-1 methylation compared to younger individuals. Across tissues, aging was consistently associated with LINE-1 hypomethylation. These findings indicate that age-related LINE-1 hypomethylation is detectable in both brain tissue and circulating cfDNA. While based on cross-sectional cohorts, the results suggest that LINE-1 methylation in cfDNA may represent a candidate minimally invasive marker of chronological aging in dogs.

**Supplementary Information:**

The online version contains supplementary material available at https://doi.org/10.1007/s11357-026-02429-y.

## Introduction

Aging is a major risk factor for disease and mortality [1] and is influenced by genetic factors. The hallmarks of aging framework originally described nine interconnected processes and was later expanded to twelve [2, 3]. Understanding the biological basis of aging and interindividual variation in aging trajectories is therefore essential. In humans, demographic aging places a substantial burden on economies and social systems, further underscoring the relevance of aging research.

At the cellular level, retrotransposons contribute to cell and tissue senescence by increasing genomic instability, which is listed as one of the primary hallmarks of aging [3]. Transposable elements make up to 46% of the human and 31% of the dog genome [4]. The most common active retrotransposons in mammals are the long interspersed nuclear element 1 (LINE-1), accounting for approximately 17% of the human genome [5, 6]. Because of the detrimental effects of excessive retrotransposon activity, multiple defense mechanisms have evolved to repress retrotransposons [7]. Much of this defense relies on heterochromatin formation, which includes histone methylation and DNA methylation. Methylation of cytosines, the covalent attachment of a methyl group to the DNA molecule, often occurs at CpG sites and plays an important role in gene expression regulation as a silencing mechanism [8]. However, age-associated changes in epigenetic regulation, including DNA methylation, have been linked to increased retrotransposon activity [9].

Following cell death, fragmented DNA is released into extracellular body fluids such as the bloodstream, where it circulates as cell-free DNA (cfDNA). The composition of plasma cfDNA reflects its tissue of origin, making it a valuable source of molecular biomarkers in clinical research. Both genetic sequence alterations and DNA methylation patterns in cfDNA are extensively studied in clinical contexts, particularly in cancer detection [10–12] and non-invasive prenatal testing [13]. LINE-1 methylation in cfDNA has also been proposed as a biomarker in several human cancers [14–16]. Beyond disease-specific applications, cfDNA may capture aspects of systemic age-related cellular turnover and epigenetic remodeling, supporting its potential utility in aging research.

Bisulfite conversion–based RT-qPCR (BSC-PCR) converts unmethylated cytosines to uracil, while methylated cytosines remain unchanged. This allows RT-qPCR with specific primers to measure their relative abundance [17]. Alternatively, MSRED-PCR employs methylation-sensitive and insensitive endonuclease digestion before RT-qPCR to estimate the methylation ratio [18]. Bisulfite conversion degrades the target DNA excessively [19, 20]. By avoiding bisulfite conversion, MSRED-PCR may reduce DNA degradation compared to bisulfite-based approaches.

Given the growing interest in translational and comparative geroscience, suitable non-human model organisms are essential for investigating molecular mechanisms of aging. The dog represents a valuable translational model for aging research. Living in close proximity to humans, dogs are exposed to many of the same environmental factors as their owners, unlike classical laboratory animals. Dogs have a shorter lifespan than humans, making longitudinal studies considerably shorter. Genetic variation is similarly extensive in dogs as in humans, and both species exhibit substantial diversity in lifespan and healthspan. Moreover, many age-related human diseases have analogues in dogs [21–23]. Therefore, dogs provide a useful model for studying aging in a naturalistic setting and represent a valuable translational system for human aging research.

LINE-1 methylation in cfDNA remains largely understudied in dogs, with existing work primarily limited to cancer-focused studies [14, 24–26]. In contrast to previous canine studies that primarily focused on disease contexts, the present study investigates LINE-1 methylation in cfDNA specifically in the context of healthy aging in dogs. Accurate characterization of disease-related phenotypes and biomarkers benefits from a clear understanding of patterns associated with healthy aging.

Here, we investigated age-associated changes in LINE-1 methylation in blood-derived cfDNA and in prefrontal cortex genomic DNA from dogs across a broad age range. Blood represents an easily accessible biological material suitable for minimally invasive assessment in clinical settings. The prefrontal cortex, in contrast, provides a central nervous system tissue with long-lived, largely postmitotic cells that are particularly relevant for studying age-related epigenetic alterations in the brain. Accordingly, LINE-1 methylation was assessed in both peripheral and neural tissues.

## Materials and methods

### Ethics statement

Blood- and brain tissue sample collection complied with national and EU law and with the institutional guidelines. The Hungarian *Animal Experiments Scientific and Ethical Committee* approved the experimental procedures under the permit number PE/EA/301–4/2021. Dog owners also provided written consent for their dogs’ participation in the study.

Brain tissue samples were obtained from owner-donated dogs euthanized for medical reasons unrelated to the present study [27].

### Sample collection and storage

#### Blood samples

Blood samples were collected from 16 Australian shepherd dogs (5 males, 11 females, 1–9 years old, Table S1) from the *vena cephalica* or *vena saphena lateralis* in accordance with the principles of *lege artis* by experienced veterinarians in the presence of the dogs’ owners. Our sample covers a broad age range of the breed, although the oldest age class was not represented. Reported average lifespan estimates range from 11 years (Australian Shepherd Health and Genetics Institute; [28]) to 12.9 years in UK VetCompass data [29]. The breed and the age of the dogs were verified using official pedigree documents and passports. Based on owners’ reports, the dogs were kept as companion dogs in Hungary, showed no signs of illness, and had not received any medications for at least two weeks prior to sampling. However, detailed control of environmental, dietary, and lifestyle factors was not possible. The animals’ health status was verified independently from the owners’ report by both a clinical examination performed by a veterinarian prior to sampling and by a veterinary diagnostic laboratory (Vet-Med-Labor Zrt., Budapest, Hungary) from whole blood and serum samples collected immediately after sampling.

#### Prefrontal cortex samples

Prefrontal cortex samples were obtained from 16 dogs of various breeds (3 beagles, 3 mongrels, 2 border collies, 2 Labrador retrievers, 1 golden retriever, 1 boxer, 1 chihuahua, 1 Gordon setter, 1 German shepherd dog, 1 Dachshund; 5 males, 11 females, 1–18 years old, Table S1). As the study relied on donations by dog owners to the Canine Brain and Tissue Bank, sample availability was constrained by the breed and age distribution of the dogs and could not be planned as precisely as for blood sampling. Two distinct age groups were defined to maximize age separation given sample availability: young dogs aged 10–50 months (n = 9) and old dogs aged 160–222 months (n = 7).

Donated cadavers were transported to the CBTB, and dissection was performed within 2–4 h *post mortem*. Whole brains were rinsed in phosphate-buffered saline for 5 min right after removal, after which prefrontal cortical samples were dissected. A 100 mg tissue sample was immersed in 1 ml RNA*later*™ Stabilization Solution (Invitrogen™, Waltham, MA USA) from each dog. Following overnight incubation at 4 °C, the supernatant was discarded, and samples were stored at − 80 °C.

### Cell-free DNA isolation

Venous blood was collected into K_3_EDTA tubes (Vacuette®, Greiner Bio-One, Kremsmunster, Austria) to avoid coagulation. Blood plasma was separated by density gradient centrifugation with Ficoll-Paque™ PLUS (Cytiva). Cell-free DNA was isolated from plasma samples using the Quick-cfDNA™ Serum & Plasma Kit (Zymo Research Corp., USA) according to the manufacturer’s protocol and stored at −80 °C until digestion.

### Genomic DNA isolation

Prefrontal cortex samples were stored at −80 °C after RNA*later*™ immersion until DNA extraction. The DNeasy Blood & Tissue kit (Qiagen, USA) was used for genomic DNA isolation according to the manufacturer’s instructions, and purified DNA samples were stored at 4 °C until downstream application.

### Primers and target sequences

Both the target LINE-1 CpG site and the primer sequences were chosen from the literature [14]. The forward primer oligonucleotide sequence was “GCTCCAGGGAGTTGGAGC” while the reverse primer sequence was “GACAGTAACAGCAACTGCG”; both sequences are reported in the 5’ → 3’ orientation relative to the positive strand. The target CpG site is located 1 bp upstream of the reverse primer binding site.

### Methylation-sensitive restriction endonuclease digestion followed by RT-qPCR

PCR reaction templates were created by using an equal input amount (1 ng) of cfDNA, which was digested at 37 °C overnight with the methylation-insensitive MspI and methylation-sensitive HpaII endonucleases (Thermo Fisher Scientific, USA). Both enzymes recognize the 5’—C^CGG—3’ sequence but differ in their ability to cleave the site depending on its methylation status. Enzymatic reactions were stopped with incubation at 65 °C (HpaII) and 80 °C (MspI) for 20 min in accordance with the manufacturers’ protocol. PowerUp™ SYBR™ Green Master Mix (Thermo Fisher Scientific, USA) was used for the real-time qPCR analysis, following the manufacturer’s instructions. The implemented PCR protocol is provided in Table S2-3. For each sample, three technical replicates were analyzed in parallel within the same run. Replicate variability was monitored across samples to ensure assay consistency.

### Bisulfite conversion followed by RT-qPCR (BSC-PCR)

We designed new BSC-PCR primers for the same CpG site, because the MSRED-PCR primers are not compatible with bisulfite-converted DNA (BSC-PCR primers are listed in Table S2). PCR templates were created using cfDNA modified by the EZ DNA Methylation-Lightning Kit according to the manufacturer’s protocol (Zymo Research Corporation, USA) to complete the bisulfite conversion and purification of the cfDNA for the methylation analysis. Due to DNA degradation associated with bisulfite treatment, this method was used primarily as an alternative assay approach for technical validation rather than for primary quantification. The methylation status of the 5′ UTR region of LINE-1 was determined by the BSC-PCR using the PowerUp™ SYBR™ Green Master Mix (Thermo Fisher Scientific, USA, Table S3-4).

Both the MSRED-PCR and BSC-PCR experiments were carried out on both the blood and prefrontal cortex tissue samples.

### Statistical analysis

#### LINE-1 sequence data analysis

A total of 264 full-length, intact canine LINE-1 sequences were available from the L1base database [30]. The longest sequence, UID199, was selected as a reference, and all other potentially active canine LINE-1 sequences were compared to it using the Needleall software from EMBOSS version 6.6.0 [31]. We then calculated the average number of mismatches in 10-bp non-overlapping windows along the entire LINE-1 sequence for each of the 263 Needleall pairwise alignments separately and then averaged the observed values. These calculations were implemented using in-house scripts.

#### Statistical modeling of the blood LINE-1 methylation levels

Every PCR experiment was repeated three times, and the technical replicates were averaged to calculate the cycle threshold (Ct) values for each observation. The differences in Ct values (ΔCt) were calculated between the methylation-insensitive and methylation-sensitive endonucleases as:

1 ΔCt=Ct(MspI)-Ct(HpaII)

Next, the exponentially transformed ΔCt value [32] was used as the response variable and modeled as a function of chronological age using the following models:

2 $${2}^{{-\Delta Ct}_{i}}={\beta}_{0}+{\beta}_{1}*{age}_{i}$$

3 $${2}^{{-\Delta Ct}_{i}}={\beta}_{0}+{\beta}_{1}*\mathrm{ln}\left({age}_{i}\right)$$

In the equation, ${\Delta Ct}_{i}$ corresponds to the differences in Ct values as described above for animal i, ${age}_{i}$ is the age of animal i (chronological age was treated as a continuous variable, measured in months) and β_0_ and β_1_ are the regression parameters. In Eq. (3), the logarithm of the age is used as the independent variable.

Similarly to the MSRED-PCR, the BSC-PCR analysis also started with averaging the three technical replicates of each of the unmethylated-specific and methylated-specific primers. These estimates then replaced the MspI’s and HpaII’s Ct values in this order in the ΔCt equation above (Eq. 1). The same regression models were fitted to the corresponding ΔCt values. All models were fitted in R, using the standard lm() function, goodness-of-fit metrics and the built-in t-test for the significance of the β_0_ and β_1_ values were used to evaluate model performance. Model assumptions were evaluated using residual diagnostics, including inspection of residual distributions.

Two samples showing extreme MSRED-PCR values, identified as influential observations in regression diagnostics and showing discordant measurements between MSRED-PCR and BSC-PCR, were excluded from the primary analysis; sensitivity analyses including these samples were also performed.

#### Statistical hypothesis testing of the prefrontal cortex methylation

Age was treated as a discrete variable with two groups (young and old) for the prefrontal cortex samples. ${\Delta Ct}_{i}$ for animal i was calculated as described above in Eq. 1 and in the text for BSC-PCR, from which ${\Delta \Delta Ct}_{i}$ was calculated for animal i as:

4 $${\Delta \Delta Ct}_{i}={\Delta Ct}_{i}-{\overline{\Delta Ct}}_{young}$$

where ${\overline{\Delta Ct}}_{young}$ is the average ΔCt value of the young age cohort, treated as a control group. Finally, a Wilcoxon rank-sum test was implemented to compare the two age cohorts’ ${2}^{-\Delta \Delta Ct}$ values [32] (normality could not be assumed, and the per-group sample size was too small for a t-test).

## Results

### LINE-1 sequence analysis

**Fig. 1:**
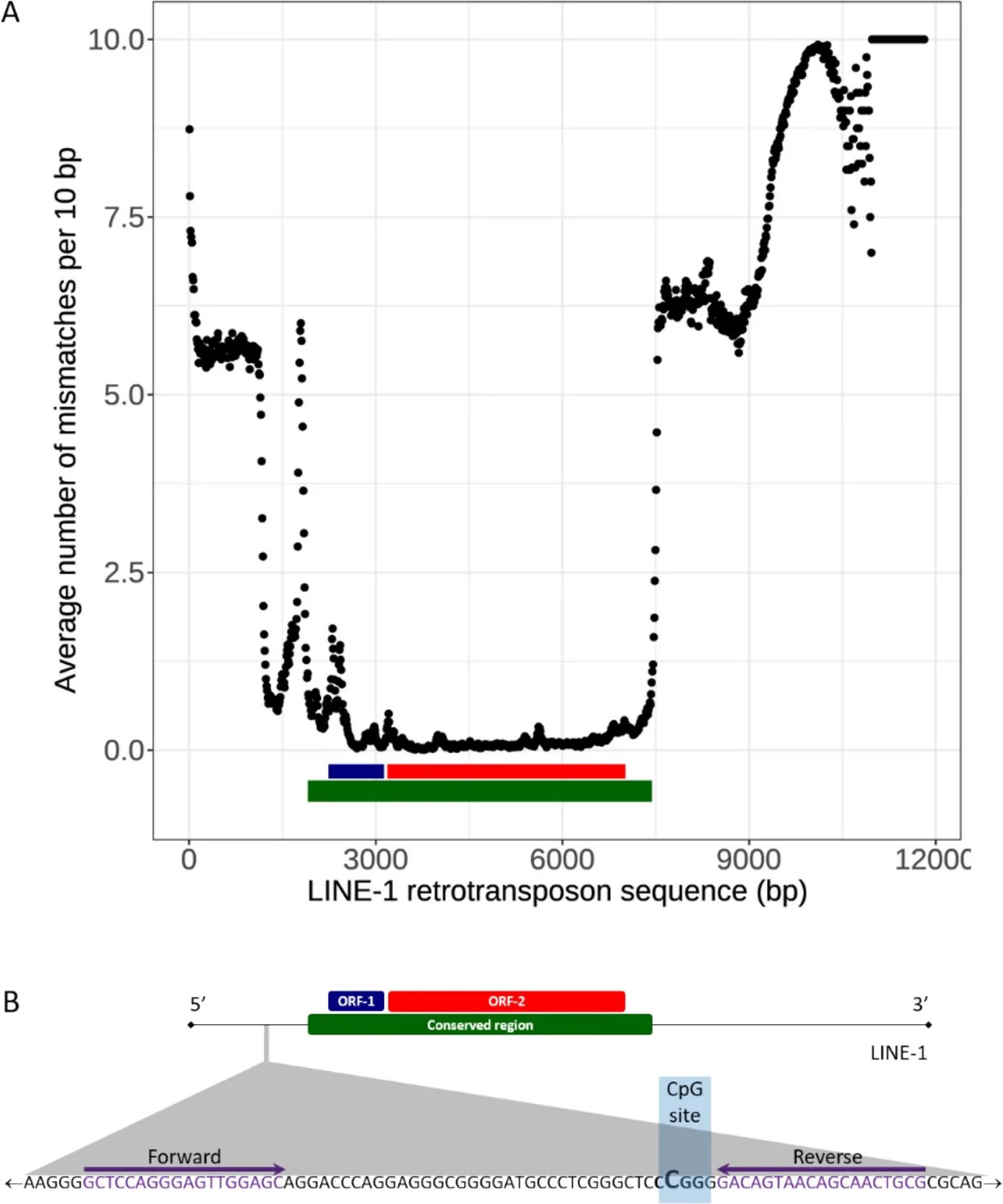
**A** Sequence similarity-based comparison of 264 potentially active canine LINE-1 elements. The green segment indicates the conserved region, while the blue and red bars denote open reading frames (ORF) 1 and 2, respectively. **B** Relative positions of the forward and reverse primers with respect to the conserved region, ORF1, ORF2 and the target CpG site within its local sequence context

A total of 264 potentially active canine LINE-1 sequences were compared based on sequence similarity. A strongly conserved region was detected in the central part of the element, spanning 5530 bp. The conserved region included both open reading frames 1 and 2 (ORF-1 and ORF-2) of the canine LINE-1 retrotransposon. The average number of mismatches across the conserved region was 0.2 bp per 10 bp. Two more variable regions flanked the conserved region at the 3’ and 5’ ends, with average mismatch rates of 8.0 and 4.2 bp/10 bp, respectively. Both the CpG site and the selected primers were located in the 5’ variable region, but in a locally less variable segment with an average of 1.04 mismatches per 10 bp (Fig. 1).

### Decreased relative CpG methylation of LINE-1

Two discordant samples were identified through a three-step assessment. First, inspection of the DCt values revealed that these two samples had DCt values approximately 4- to 7-fold higher values than samples from similarly aged dogs. However, because the three technical PCR replicates within each sample were highly consistent, the samples were retained for initial modelling. Second, regression diagnostics of the model including all samples identified these observations as outliers and influential points (data not shown). Inspection of the individual Ct measurements indicated that the extreme DCt values were driven primarily by the MspI measurements in these two samples (CF_blood_02 and CF_blood_04). Finally, BSC-PCR analysis of the same samples did not identify them as outliers within their respective age groups (Fig. S1; the two samples are marked with red dots in the logarithmic model plot). Taken together, the extreme MSRED-PCR values, their influence on the regression model, and the discordance between the MSRED-PCR and BSC-PCR results supported treating these observations as assay-specific technical outliers. They were therefore excluded from the primary analysis.

**Fig. 2:**
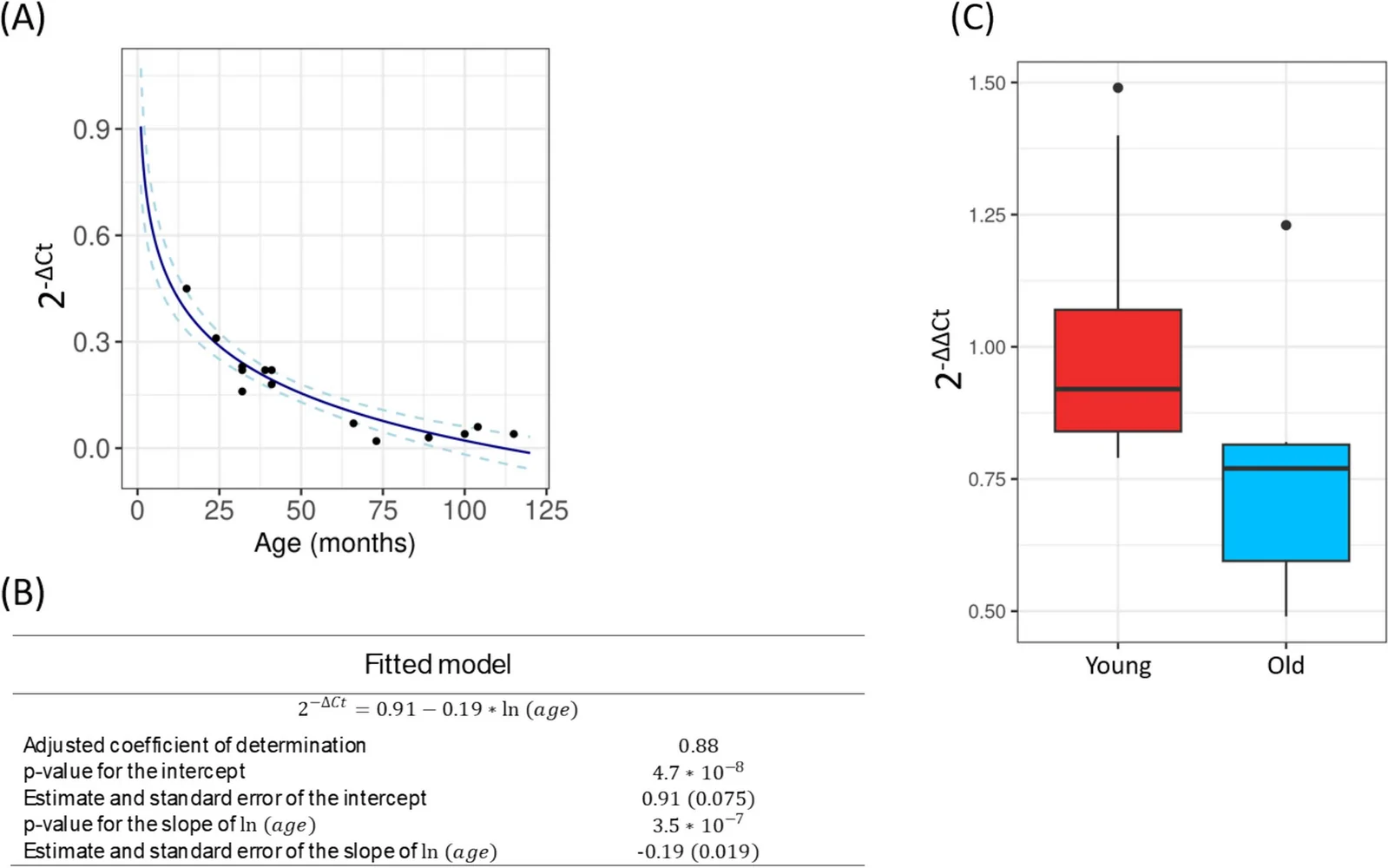
Output of methylation-sensitive restriction endonuclease digestion coupled with PCR (MSRED-PCR). **A** Relative methylated-to-unmethylated proportions (expressed as 2^−ΔCt^), plotted as a function of chronological age (in months) of 14 blood samples from dogs of the same breed (Australian shepherd dog). The best-fitting logarithmic model (solid line) and its 95% confidence interval (dashed lines) are shown. **B** Model-fitted values derived from the logarithmic regression applied to the blood MSRED-PCR data. **C** Relative methylated-to-unmethylated proportions (expressed as 2^−ΔΔCt^) in two age groups derived from prefrontal cortex samples

We observed a strong negative correlation between age and methylation (Spearman’s rho = −0.86; Fig. 2A). Both linear and logarithmic models were fitted to the observed data to model the relationship between age and methylation status of the studied CpG site (parameter estimates, their 95% confidence intervals, the adjusted R^2^ and mean square errors are presented in Table S5). The logarithmic model provided a better fit than the simple linear regression based on goodness-of-fit metrics (Fig. 2, Fig. S2). Indeed, the adjusted coefficient of determination (adjusted R^2^) was higher with the logarithmic model than with the linear model (0.89 vs. 0.72), while the residual standard error was smaller (0.043 with the logarithmic model, 0.068 with the linear model). Both the intercept and the age-dependent term were statistically significant (p < 0.001) in both models. Distributions of the residuals were acceptable for both models (Fig. S3), while mean square errors were 0.0039 and 0.0016 for the linear and logarithmic models, respectively.

As a sensitivity analysis, we refitted the logarithmic model with the two excluded samples included. The analysis retained a negative association between age and LINE-1 methylation levels (Fig. S1), consistent with the primary analysis. Although inclusion of the two samples resulted in wider confidence intervals, the direction of the age effect remained unchanged, indicating that the main conclusion was robust to their inclusion. The same conclusion was obtained for the linear model (Table S5).

The genomic DNA analysis from the prefrontal cortex samples also showed a similar direction of effect: a Wilcoxon rank-sum test was used to test the hypothesis that the $\Delta \Delta Ct$ values differed significantly between the two age cohorts (W = 54, p = 0.016, Fig. 2C).

## Discussion

We analyzed 264 potentially active canine LINE-1 retrotransposon sequences and identified a highly conserved central region encompassing both open reading frames, flanked by more variable regions at the 3′ and 5′ ends. Although sequence variability was pronounced in the flanking regions, the CpG site targeted in this study was located within a locally less variable segment of the 5′ region, exhibiting one of the lowest mismatch rates observed (1.04 bp per 10 bp). The low sequence variability at the primer binding sites suggests that the applied assays likely detect signals derived from multiple LINE-1 copies rather than a single locus. Accordingly, our findings are interpreted as aggregate LINE-1 methylation dynamics across multiple LINE-1 copies.

DNA methylation levels vary substantially between mammalian species [33], underscoring the importance of species-specific characterization. In our cohort, LINE-1 methylation in blood cfDNA showed a strong age-dependent decline, best captured by a nonlinear trajectory in the MSRED-PCR experiment. The use of a single-breed cohort minimized interbreed genetic heterogeneity and reduced potential genetic confounding, although at the expense of lower generalizability of the results to the entire species. An alternative analysis applying the BSC-PCR procedure showed a similar direction of effect, consistent with the age-related LINE-1 hypomethylation observed with the MSRED-PCR method.

In the prefrontal cortex cohort, chronological age was treated as a categorical variable, and donors were stratified into young and old groups separated by approximately ten years. The mixed-breed composition and discrete age design reflect the practical constraints of donor-based tissue collection. The MSRED-PCR experiment on prefrontal cortex samples provided further support for the age-related decline of LINE-1 methylation observed in the more accessible blood tissue. Substantial within-group variability was observed in both the blood and the prefrontal cortex BSC-PCR data (Fig. 2C and S4), which may partly arise from methodological limitations, as bisulfite conversion is known to induce DNA degradation [20].

Due to practical constraints, the experimental design differed significantly between the blood and prefrontal cortex experiments. While a single-breed experiment could be designed and implemented in the case of the more accessible blood tissue, the experiments on prefrontal cortex depended on owner donations to the Canine Brain and Tissue Bank, and consequently a multi-breed analysis had to be implemented. These differences in study design limit direct comparisons between the blood and prefrontal cortex findings and restrict the generalizability of the results across breeds, tissues, and health conditions. External validation in larger and more diverse cohorts will therefore be necessary.

Vershinina et al. showed that age-associated DNA methylation changes do not necessarily follow linear trajectories across the lifespan and highlighted the importance of incorporating nonlinear models into epigenetic aging research [34]. Consistent with these observations, our findings suggest that a logarithmic model may more accurately capture the relationship between chronological age and LINE-1 methylation than a linear model. The estimated mean square errors were 2.5 times higher in the linear model than in the logarithmic model, indicating a better fit with the latter. In accordance with these findings, the residual diagnostics showed a better fit with the logarithmic model (Fig. S3). This further supports the broader applicability of nonlinear approaches in methylation-based aging analyses. However, given the modest sample size and single-breed composition of the blood cohort, the logarithmic model should be considered exploratory and cohort-specific rather than evidence of a universal nonlinear trajectory of age-related LINE-1 methylation.

Our results further suggest that age-related epigenetic alterations extend beyond gene-associated regions to include active retrotransposons [35, 36]. We hypothesize that progressive LINE-1 hypomethylation may reflect reduced epigenetic repression with advancing aging and could facilitate increased retrotransposon activity. However, direct assessment of LINE-1 mobilization will be necessary to determine whether the observed hypomethylation translates into de novo insertions at LINE-1 target sites.

DNA methylation clocks have recently been developed for dogs [37] primarily using genomic DNA from peripheral blood or tissue samples. Our findings extend this framework by suggesting that chronological age can be modeled using plasma-derived cfDNA methylation. Using an MSRED-PCR approach, we observed a strong association between LINE-1 methylation and age, suggesting that cell-free DNA-based methylation measures track age-related variation within this cohort. Whether cfDNA methylation also reflects biological age remains to be determined.

A limitation of the present study is the cross-sectional design, the modest sample size, and the single-breed composition of the blood cohort, which may limit generalizability. Future studies in larger and more diverse populations are needed to determine whether cfDNA LINE-1 methylation reflects not only chronological but also biological age in dogs.

## Conclusions

We show that LINE-1 methylation in plasma-derived cell-free DNA and prefrontal cortex genomic DNA is associated with chronological age in dogs. The relationship is best described by a nonlinear (logarithmic) model, consistent with age-related hypomethylation. These findings support the potential utility of LINE-1 CpG methylation as a candidate minimally invasive marker of chronological aging in dogs. Further validation in larger and more diverse cohorts will be required to establish its robustness and generalizability.

Supplementary information.

## Supplementary Information

Below is the link to the electronic supplementary material.

Supplementary file1 (PDF 265 kb)

## Data Availability

Raw LINE-1 sequence data is publicly available online in the L1Base database [30]. All log files and scripts used for data analysis are publicly available on *Science Data Bank* (https://www.scidb.cn) with the following DOI accession number: https://doi.org/10.57760/sciencedb.38528. Raw PCR data and input data for the models are available on *Science Data Bank* under the following DOI accession number: https://doi.org/10.57760/sciencedb.38653.
