# Supplementary material for: LINE-1 CpG methylation in canine blood and cortical DNA tracks epigenetic changes with age
Source: GeroScience. 2026 Jul 27;48(5):6223–32. doi: 10.1007/s11357-026-02429-y (PMC13601471; doi:10.1007/s11357-026-02429-y)

Supplementary Material

Table S1: Demographic information of the dogs

Tissue	Dog ID	Breed	Sex	Age (months)
Blood	CF_blood_01	Australian shepherd	Female	15
	CF_blood_02	Australian shepherd	Male	23
	CF_blood_03	Australian shepherd	Female	24
	CF_blood_04	Australian shepherd	Female	27
	CF_blood_05	Australian shepherd	Female	32
	CF_blood_06	Australian shepherd	Female	32
	CF_blood_07	Australian shepherd	Female	32
	CF_blood_08	Australian shepherd	Female	39
	CF_blood_09	Australian shepherd	Male	41
	CF_blood_10	Australian shepherd	Male	41
	CF_blood_11	Australian shepherd	Female	66
	CF_blood_12	Australian shepherd	Female	73
	CF_blood_13	Australian shepherd	Male	89
	CF_blood_14	Australian shepherd	Male	100
	CF_blood_15	Australian shepherd	Female	104
	CF_blood_16	Australian shepherd	Female	115
Brain - prefrontal cortex	CF_brain_01	Border collie	Female	10
	CF_brain_02	Boxer	Female	16
	CF_brain_03	Chihuahua	Female	16
	CF_brain_04	Beagle	Female	16
	CF_brain_05	Beagle	Female	14
	CF_brain_06	Beagle	Male	47
	CF_brain_07	Gordon setter	Female	50
	CF_brain_08	German shepherd	Male	46
	CF_brain_09	Mongrel	Male	24
	CF_brain_10	Labrador retriever	Male	165
	CF_brain_11	Labrador retriever	Female	174
	CF_brain_12	Border collie	Female	169
	CF_brain_13	Golden retriever	Female	212
	CF_brain_14	Dachshund	Female	205
	CF_brain_15	Mongrel	Female	201
	CF_brain_16	Mongrel	Male	222

Table S2. RT-qPCR primers used for bisulfite conversion–based PCR (BSC-PCR) targeting canine LINE-1. The 3′ terminal nucleotide of each reverse primer (highlighted in red) corresponds to the interrogated CpG site. Either L1_BSC_reverse or L1_BSC_reverse_Met was used depending on the methylation status of the template.

Primer name	Specificity	Sequence (5'→3')
L1_BSC_forward	Universal	ATA AAG GTT TTT AAG GGG GAG GGG TTT TGT GAG
L1_BSC_reverse	Unmethylated-specific	ACA CAC TCT CCT ACA CAC AAT TAC TAT TAC TAT CC**C CA**
L1_BSC_reverse_Met	Methylated-specific	ACA CAC TCT CCT ACA CAC AAT TAC TAT TAC TAT CC**C CG**

Table S3. Reaction mixtures used for MSRED-PCR and BSC-PCR assays

Component	MSRED PCR (µL)	BSC PCR (µL)
PowerUp SYBR Green Master Mix	5	5
Ultra Pure Water	2	2
Forward Primer	0.5	1
Reverse Primer	0.5	1
Template	2	1
Total	10	10

Table S4. Thermal cycling conditions for MSRED-PCR and BSC-PCR

Step	Temperature (°C)	Duration (sec)	Ramp (°C/sec)
Preincubation	50	120	4.4
Initial denaturation	95	120	1.0
Amplification (MSRED-PCR: 40 cycles; BSC-PCR: 60 cycles)			
Denaturation	95	15	2.2
Annealing	60	15	2.2
Extension (single acquisition)	72	60	4.4
Final extension	72	600	2.2

Table S5: Regression parameter estimates and model fit parameters.

Model type		Intercept (β_0)		Slope (β_1)		adjusted R^2	MSE[1]
		Estimate	95% CI	Estimate	95% CI		
Logarithmic	Including outliers	0.659	[0.308; 1.011]	-0.137	[-0.228; -0.045]	0.38	0.009
	Excluding outliers	0.917	[0.753; 1.082]	-0.195	[-0.237; -0.153]	0.89	0.002
Linear	Including outliers	0.267	[0.154; 0.380]	-0.002	[-0.004; -0.001]	0.31	0.010
	Excluding outliers	0.348	[0.269; 0.430]	-0.003	[-0.005; -0.002]	0.72	0.004

1: mean square error

Figure S1. Output of bisulfite conversion–based RT-qPCR (BSC-PCR). Relative methylated-to-unmethylated proportions plotted as a function of chronological age (in months) in 16 blood samples from dogs of the same breed. The plotted $2^{-\Delta Ct}$ parameter can be interpreted as the relative ratio of methylated to unmethylated cell-free DNA. Left: the best-fitting linear model (blue line) with its 95% confidence interval (dashed blue lines) is shown. Right: the best-fitting logarithmic model is shown in blue, with its 95% confidence interval (dashed lines). The two samples identified as outliers in the MSRED-PCR analysis are indicated with red dots. These samples were not outliers in this BSC-PCR analysis and were therefore interpreted as technical outliers specific to the MSRED-PCR assay.

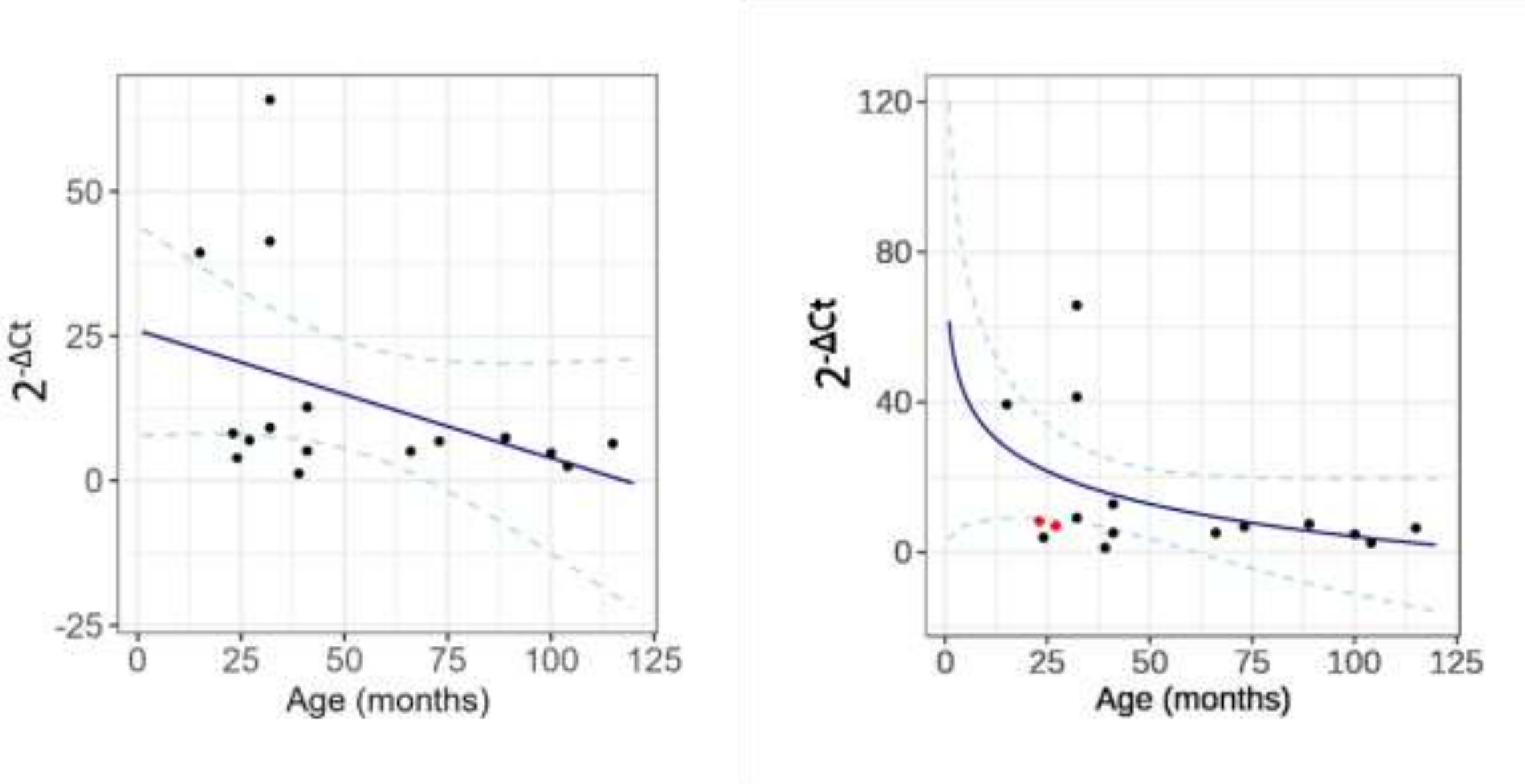

Figure S2. Linear model fitted to the methylation-sensitive restriction endonuclease digestion coupled with PCR results obtained from the blood samples. Relative methylated-to-unmethylated proportions (expressed as $2^{-\Delta Ct}$) were modelled as a function of the chronological age of the dogs (in months). The 95% confidence interval is indicated by dashed lines. Regression parameters: $\beta_0 = 0.348$ (t-test p-value: 6.3×10^{-7}), $\beta_1 = -0.003$ (t-test p-value: 7.9×10^{-5}), adjusted $R^2 = 0.72$, and residual standard error = 0.068.

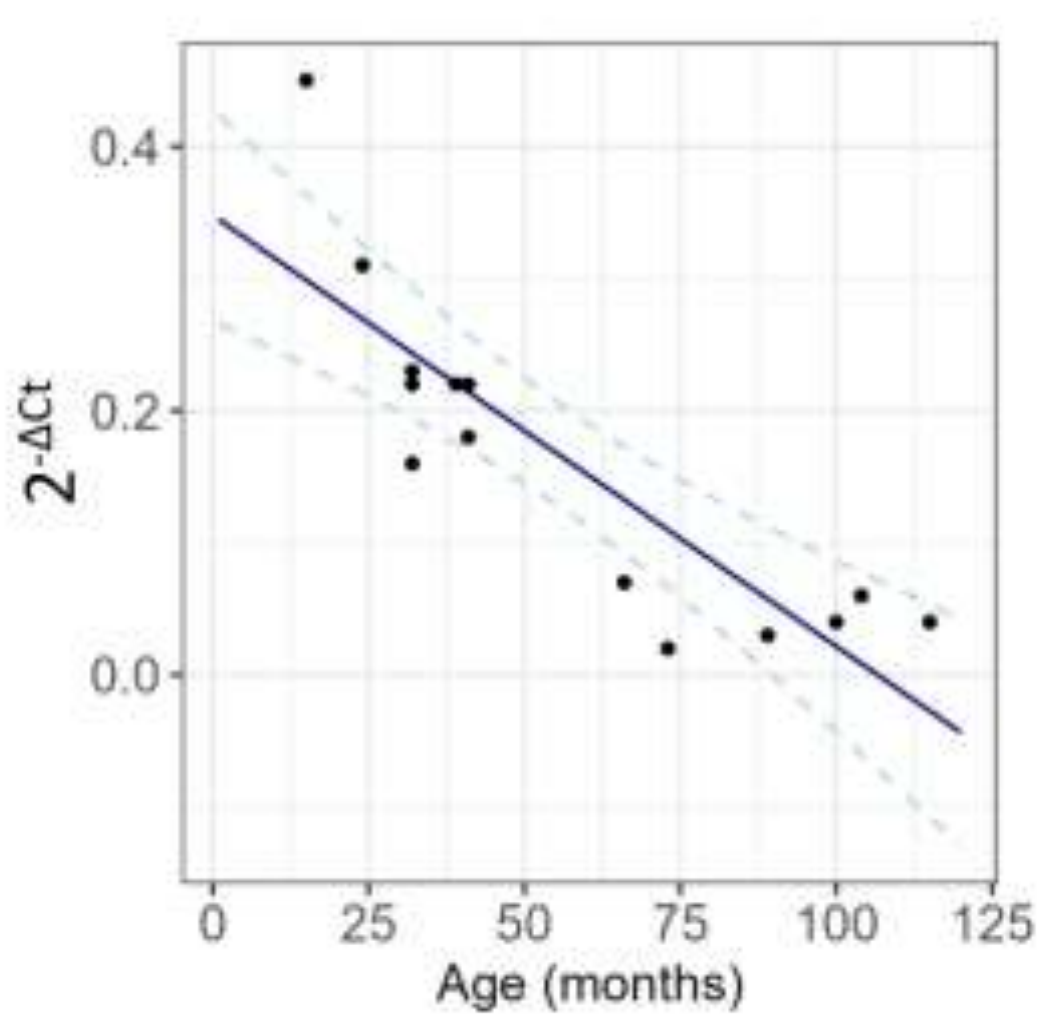

Figure S3. Residual diagnostics plots for the linear (A) and logarithmic (B) models fitted to the methylation-sensitive restriction endonuclease digestion-PCR results. Original models are shown on Figure S2 (linear model) and Figure 2 (logarithmic model).

A **B**

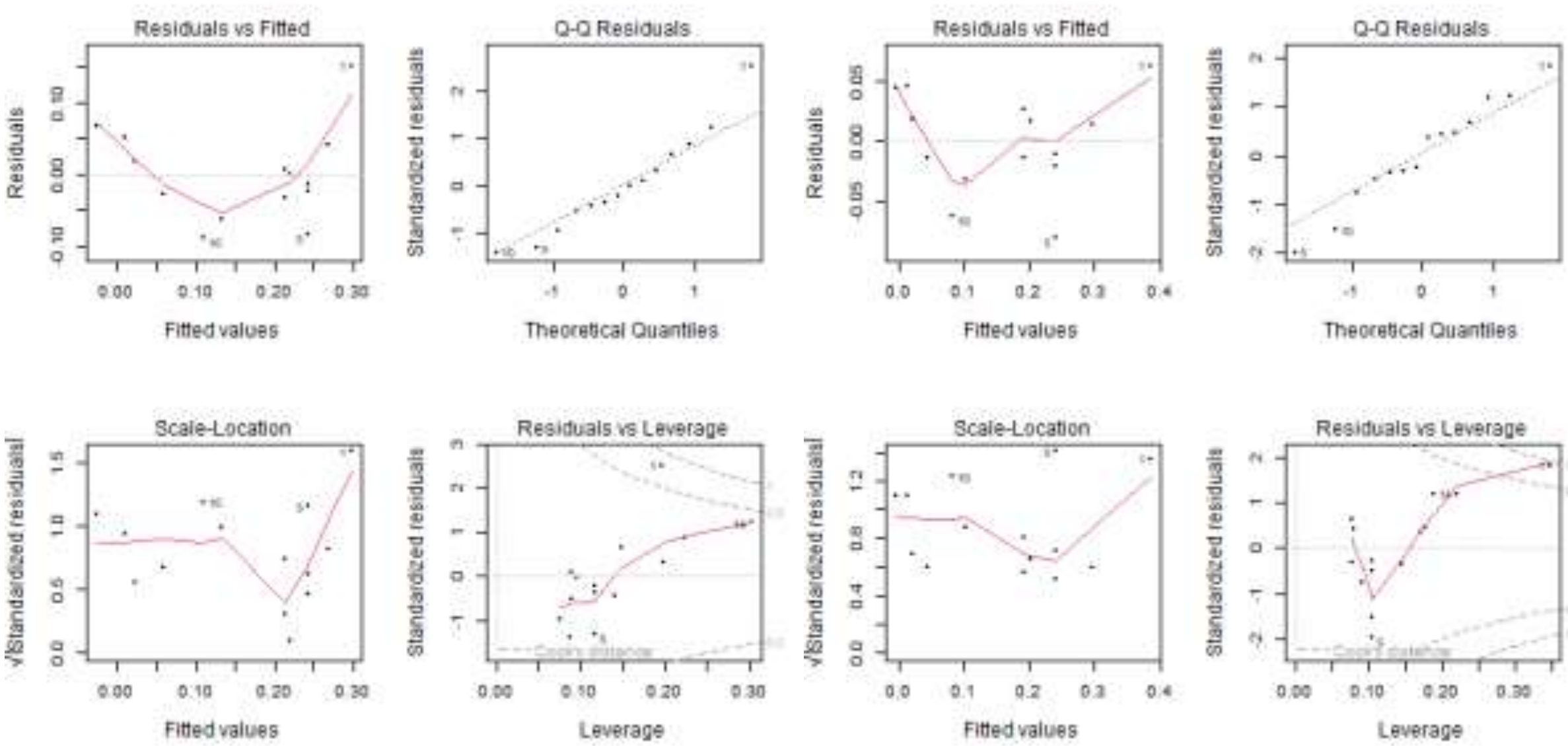

Figure S4. Output of bisulfite conversion–based RT-qPCR (BSC-PCR). Relative methylated-to-unmethylated proportions in two age groups derived from prefrontal cortex samples. The plotted $2^{-\Delta\Delta Ct}$ parameter represents the relative methylated to unmethylated DNA ratio. No significant difference between age groups was detected (Wilcoxon rank-sum test, W = 19; p = 0.39). Dots indicate outliers.

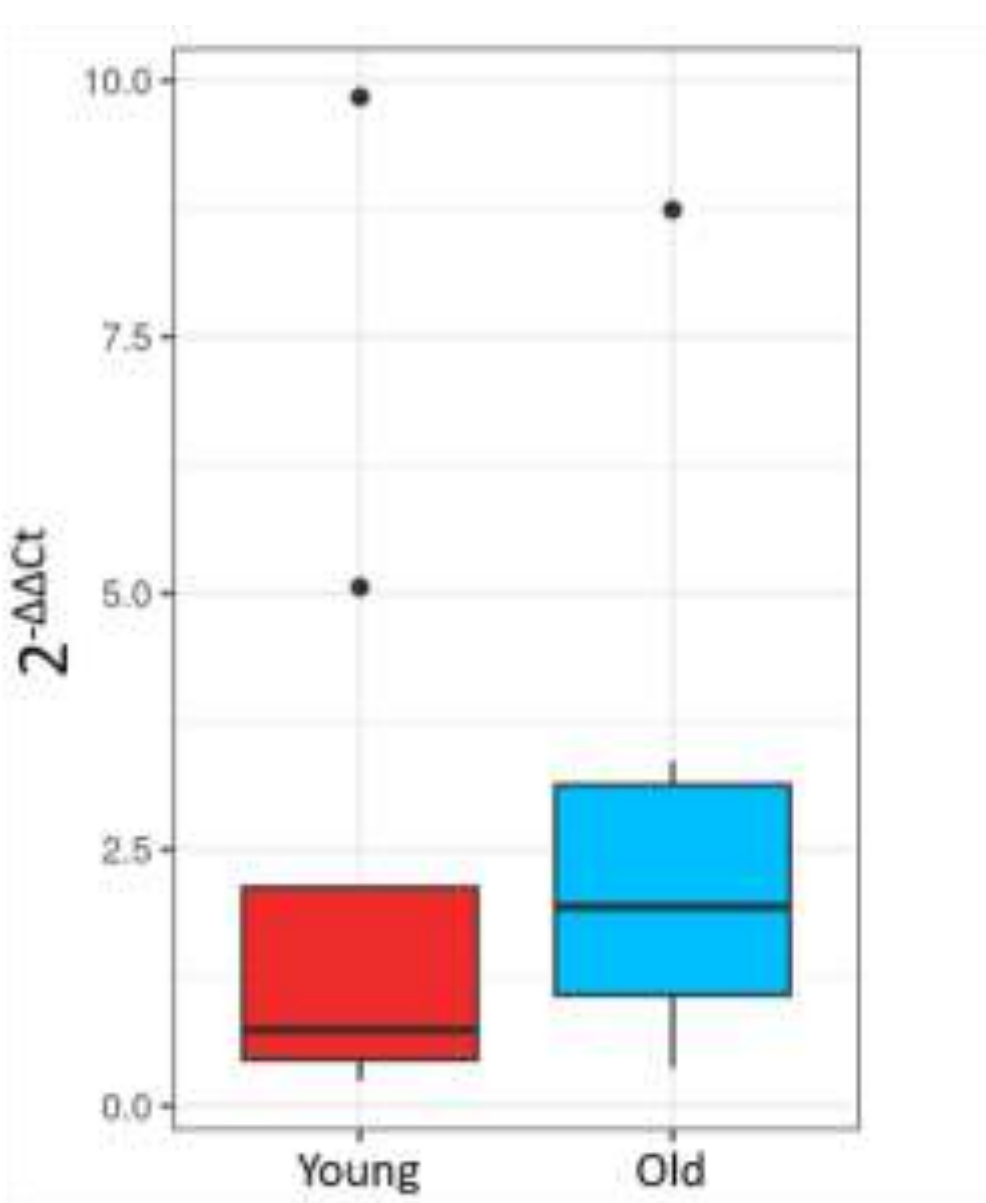

Supplement: Supplementary file 1 [file 11357_2026_2429_MOESM1_ESM.pdf]
